# Hypoxia after stroke: a review of experimental and clinical evidence

**DOI:** 10.1186/s13231-016-0023-0

**Published:** 2016-12-07

**Authors:** Phillip Ferdinand, Christine Roffe

**Affiliations:** 1 The Royal Wolverhampton NHS Trust, Wolverhampton, WV10 0QP UK; 2 Stroke Research in Stoke, Institute for Applied Clinical Studies, Keele University, Keele, Staffordshire UK

**Keywords:** Cerebral hypoxia, Acute stroke, Oxygen therapy, Oxygen physiology, Cerebral blood flow

## Abstract

**Background:**

Hypoxia is a common occurrence following stroke and associated with poor clinical and functional outcomes. Normal oxygen physiology is a finely controlled mechanism from the oxygenation of haemoglobin in the pulmonary capillaries to its dissociation and delivery in the tissues. In no organ is this process more important than the brain, which has a number of vascular adaptions to be able to cope with a certain threshold of hypoxia, beyond which further disruption of oxygen delivery potentially leads to devastating consequences. Hypoxia following stroke is common and is often attributed to pneumonia, aspiration and respiratory muscle dysfunction, with sleep apnoea syndromes, pulmonary embolism and cardiac failure being less common but important treatable causes. As well as treating the underlying cause, oxygen therapy is a vital element to correcting hypoxia, but excessive use can itself cause molecular and clinical harm. As cerebral vascular occlusion completely obliterates oxygen delivery to its target tissue, the use of supplemental oxygen, even when not hypoxic, would seem a reasonable solution to try and correct this deficit, but to date randomised clinical trials have not shown benefit.

**Conclusion:**

Whilst evidence for the use of supplemental oxygen therapy is currently lacking, it is vital to rapidly identify and treat all causes of hypoxia in the acute stroke patient, as a failure to will lead to poorer clinical outcomes. The full results of a large randomised trial looking at the use of supplemental oxygen therapy are currently pending.

## Background

Hypoxia is common after stroke, and associated with poor outcomes. In this article, we have reviewed the physiology of oxygen transport, the cerebrovascular response to hypoxia and pathophysiology, incidence and aetiology behind hypoxia in stroke and its subsequent clinical consequences. We have then reviewed all randomised clinical trials looking at the use of supplemental oxygen therapy in acute stroke and made conclusions regarding current evidence and recommendations for clinical practice.

## Oxygen physiology

The normal adult range of arterial oxygen pressure (PaO2) is 11.0–14.4 kPa and the normal range for arterial oxygen saturation (SaO2) is 95–98% [1]. The term hypoxia refers to oxygen levels below normal. It includes both tissue (e.g. brain, myocardium) hypoxia and hypoxia in the blood (hypoxaemia). Tissue hypoxia is defined by the concentration of oxygen in blood and also tissue perfusion, whilst hypoxaemia is defined by the concentration of oxygen in inspired air and its transfer into the blood [2].

Following inhalation oxygen is taken up in the lung capillaries via diffusion down an oxygen concentration gradient across the alveoli [3, 4]. Oxygen binds to the haemoglobin molecule, which can carry four oxygen molecules; each binding and changing the shape of the haemoglobin molecule and increasing its affinity for oxygen [5]. A small amount of oxygen is also dissolved in plasma. This proportion increases in hyperoxia, when all haemoglobin is saturated [6]. Oxygen dissociates from the haemoglobin molecule in the tissues owing to the relatively hypercapnic and acidic environment (the Bohr effect) [3].

Oxygen is a vital substrate that supports virtually all metabolic processes. 90% of oxygen intake is engaged in the cytochrome C oxidase system in the mitochondria [7] generating adenosine triphosphate (ATP), which acts as the main energy substrate within cells. A continuous supply of oxygen is required to secure a continuous supply of ATP maintaining sufficient energy for cerebral neuronal and cellular activity. This facilitates an efficient energy producing process making 38 molecules of ATP during aerobic respiration, equivalent to 1270 joules (J) energy, in comparison to two molecules of ATP (67 J of energy) during anaerobic respiration [7, 8].

## The anoxic brain

20% of all human oxygen consumption is utilised by the brain [9]. The brain has no oxygen or glucose (the other important substrate in the ATP producing equation) stores. Thus complete disruption of cerebral blood flow very rapidly results in an anoxic, hypoglycaemic state, which via a variety of mechanisms ultimately leads to cell death. Excitatory neurotransmitters, such as glutamate, bind to a variety of receptors and allow for an influx of calcium ions that help formulate the chemical signal for depolarisation [10, 11]. Normally the re-uptake of glutamate is an active energy-driven process. In the absence of ATP this process fails, resulting in an extracellular accumulation of glutamate, which continually stimulates receptors leading to a persistent influx of calcium ions [12]. Furthermore, the Na+/Ca2+ ATP driven pump normally used to eliminate calcium fails, also due to a lack of ATP [13]. The resultant high intracellular calcium triggers multiple cascades that ultimately lead to mitochondrial dysfunction and cell death. Furthermore, instead of producing ATP, glial cells have been shown to release ATP extracellularly [11]. Aside from rendering this unusable by mitochondria, ATP also stimulates the P2X7 receptor, which again leads to significant calcium influx and ultimately cell death [13]. The other major mechanism of cellular demise is via the formation of free radicals facilitated by the reduction of iron from its ferric (Fe3+) to its ferrous (Fe2+) form and the initiation of inflammatory cascades [12].

## Cerebral blood flow in hypoxia

In normoxic states, cerebral blood flow is very tightly controlled by the partial pressure of carbon dioxide (PaCO_2_). Any hypocapnic state will result in vasoconstriction and reduction in regional cerebral blood flow and a hypercapnic state leads to the reverse with vasodilatation and an increase in cerebral blood flow. Cerebral blood flow is somewhat less responsive to changes in PaO_2_, which has the opposite effect to carbon dioxide; a hypoxic state causing cerebral vasodilatation with the aim of improving oxygen delivery and a hyperoxic state causing vasoconstriction [14, 15].

In a hypoxic state, whilst the vasodilatory response improves flow, the detection of hypoxia by peripheral chemoreceptors will in turn lead to an increase in respiratory drive, increasing arterial oxygen content. However, the consequence of this is also an increase in the clearance of carbon dioxide, which would theoretically cause vasoconstriction and reduced cerebral blood flow [9]. It appears there is a threshold to which the hypoxic response predominates (and the carbon dioxide one attenuated) at a PaO_2_ of around 50–60 mmHg [9, 14]. Whilst the carbon dioxide mediated vascular response is mediated via a direct change in vessel wall pH [15], the oxygen response appears to be mediated by the deoxygenated erythrocyte via a number of mechanisms; which include release of ATP and the subsequent actions of endothelial nitric oxide synthase on the vessel wall, reduction of nitrite to nitric oxide and the activity of S-nitrosohaemoglobin [9].

The cerebral vascular response to hypoxia is not uniform. A study found that in an induced isocapnic hypoxic state increases in cerebral blood flow were most prominent in basal ganglia nuclei, the putamen, thalamus, nucleus accumbens and pallidum [16]. Studies of blood flow in individual vessels have found that flow in the internal carotid artery is maintained during hypoxia and that vertebral artery flow is increased [17]. This had led to the hypothesis that blood flow is increased in this region to preserve vital brainstem structures, or that possibly the posterior circulation vasculature is less susceptible to the effects of carbon dioxide for similar reasons.

## Neurological effects of hypoxia

The neurological consequences of hypoxia are dependent upon the speed of onset, the severity of hypoxia, and the level of tissue perfusion. Rapid decreases in PaO_2_, as in a cardiorespiratory arrest, can lead to permanent neurological damage within minutes. However, lower, less abrupt changes, can be tolerated if the decrease in oxygen occurs in a gradual manner, such as ascending at altitude, where individuals can acclimatise and develop tolerance to lower oxygen partial pressures or, (to a lesser degree), in chronic smokers. Initial clinical features include altered judgement, difficulty in completing complex tasks, and impairment in short term memory [18, 19], but in the longer term deficits can be more widespread and span physical and neuropsychological domains. Seizures occur in up to a third of individuals within a day of exposure to hypoxia, and are commonly partial complex or myoclonic in nature. Intractable forms of either of these types of seizure are associated with a poor prognosis [20]. Cognitive impairment domains include amnesia, visuospatial deficits, frontal lobe symptoms, impairment of executive function, and impairments in language [21]. These are covered in more detailed reviews on the subject [22, 23]. Involvement of the basal ganglia, a region particularly susceptible to hypoxic injury, can result in delayed Parkinsonism in older subjects, dystonia mainly in younger people, choreo-athetosis, and tremors [20]. Varying degrees of unilateral or bilateral motor impairment may be observed depending on both the anatomical level and extent of corticospinal tract involvement. In very rare cases, the syndrome of delayed post-hypoxic leukoencephalopathy may occur weeks after a seemingly rapid recovery from the original insult. This condition is characterised by rapid deterioration in cognition, emergence of extra-pyramidal signs, and loss of executive function as a results of severe demyelination [24]. The severity of leukoencephalopathy can be assessed by magnetic resonance imaging (MRI) [20]. Electroencephalography, somatosensory evoked potentials and MRI can provide valuable information about the severity of hypoxic injury, but also aid in prognostication together with overall clinical state [25].

## Hypoxia in the context of a stroke

There is no specific definition as to what constitutes hypoxia in an acute stroke, and it is therefore reasonable to assume that normal values for the general population apply.

Sulter and colleagues [26] monitored 49 consecutive patients who presented with an acute stroke within 12 h duration using pulse oximetry for 48 h. Patients were considered hypoxic and treated with supplemental oxygen if saturations were below 96% for more than 5 min. This occurred in 63% [31] of patients, with 28 of those returning to ‘normal’ oxygen saturations following administration of up to 5 L/min of oxygen. The remaining three required much higher concentrations. Factors associated with hypoxia in this group were stroke severity, presence of dysphagia, and older age. Roffe et al. [27] recruited 118 patients (100 of whom had adequate measurements by pulse oximetry) and found that the mean daytime awake SO_2_ was 94.5 ± 1.7% in stroke patients and 95.8 ± 1.7% in healthy controls. Nocturnal saturations were reduced to 93.5 ± 1.9% in the stroke group and 94.3 ± 1.9% in controls. In the stroke group the average 4% oxygen desaturation index (ODI) (number of times per hour the saturation dipped more than 4% from baseline) was higher than in controls. At night almost a quarter of the stroke group had desaturations below 90%. The same group also looked further at the differences between day and night oxygen saturations [28]. In stroke patients who were not hypoxic (defined as SaO_2_ less than 90%) during the day, baseline daytime saturations were measured between 9am and 9pm and nocturnal saturation between 10pm and 6am. In total 40 patients were recruited and in addition to SaO_2_, respiratory rate and sleep/awakeness was measured twice in each time period. The mean respiratory rate day vs. night was 20 and 18 breaths per minute respectively. The mean daytime SaO_2_ was 95.5% (87–98.6%) and 94.3% (80–98%) at night. There was a strong correlation between respiratory rate, SaO_2_ and the 4% ODI, making it clear that borderline daytime hypoxia could predict nocturnal hypoxic episodes. Comparisons in a later study were then made with matched controls overnight [29]. In this study the mean nocturnal oxygen saturations were found to be 0.5% less than controls, with the lowest measured desaturation in this group of 79.4%, still almost 6% lower than the control group. The largest difference was in the percentage of patients with more than 10 desaturations per hour (42% stroke vs. 15% controls). Hand et al. [30] performed a study looking at the feasibility of MRI as an imaging modality in hyperacute stroke assessment. One of the eligible 138 patients for the study could not be scanned owing to pulmonary oedema severe enough to cause considerable hypoxia. For a variety of reasons it was only possible to consistently measure oxygen saturations in 61 out of 85 patients. In those in whom saturations could reliably be measured, 11 out of 61 developed hypoxia (lowest 74%) and of those who received oxygen during the scan only two could be monitored successfully. This highlights not only the prevalence of hypoxia in acute stroke, but the logistical difficulties acute hypoxia may pose for assessment. Another study examined the effect of five different, but randomly ordered body positions, each for 10 min on the impact on oxygen saturation [31]. Interestingly, lying on the left hand side reduced oxygen saturations, but only in those who hand a right hemiparesis. Those who were able to sit in a chair were able to achieve much higher mean SaO_2_, albeit suffering from more minor strokes. It was felt that a severe stroke, with a right hemiparesis and underlying chest disease were the greatest predictors of desaturation, but only when lying on the left side. A subsequent systematic review [32] comprising of three randomised controlled trials (173 patients) and one case controlled trial (10 patients) found that body position only played a role in oxygen saturations if patients had underlying respiratory co-morbidities.

The risk of aspiration is well documented in acute stroke (see below), but independent of this the question as to whether feeding (oral or nasogastric) contributes to hypoxia has also been examined. Dutta et al. [33] reported that nasogastric feeding caused no decrease in SaO_2_. A later study [34] found a small but statistically non-significant trend towards hypoxia when tube fed, in particular in patients that were fed overnight. Rowat and colleagues looked at the impact of oral feeding on oxygen saturations, using hospitalised elderly patients and young healthy controls as comparators [35]. The baseline SaO_2_ was lower in the stroke cohort than the other two, with a very small decrease in SaO_2_ with oral feeding in the stroke (0.1%) and the elderly groups. Nearly a quarter of stroke patients dropped SaO_2_ to less than 90% (16% elderly, 0% young), but this did not occur in close relation to the time of swallowing, and thus no immediate risk could be attributed to oral feeding.

There is relatively little research on the correlation between hypoxia and clinical outcome. Hypoxia has been shown to be an independent clinical risk factor for post stroke dementia [36]. Rowat et al. [37] found that hypoxic patients were more likely to have respiratory disease and this led to an increased mortality. A smaller study looked at the prevalence of hypoxia in patients undergoing rehabilitation and found no significant difference in mean SaO_2_ at baseline, in nocturnal SaO_2_, the lowest nocturnal SaO_2_ or in the 4% ODI [38]. In conclusion, no association between SaO_2_ and functional outcome was found. Hypoxia has, however, been shown to correlate with the degree of white mater disease on MRI. White matter hyperintensity volumes were greatest in obstructive sleep apnoea (OSA) patients compared with non-OSA patients and more explicitly in hypoxic compared to non-hypoxic patients [39].

## Causes of hypoxia in acute stroke

Pneumonia is a frequent complication of acute stroke. A recent consensus defined the term stroke-associated pneumonia (SAP) as a representation of a spectrum of lower respiratory tract disorders occurring within 7 days after the onset of stroke [40]. The criteria were based on a modified version of the centre for disease control (CDC) criteria, with a probable SAP fulfilling all CDC criteria but not meeting typical chest radiography changes and definite SAP fulfilling all CDC criteria including typical chest X-ray changes. In addition the consensus group concluded that there was a limited role for C-reactive protein, white blood cell count and other inflammatory biomarkers in the diagnosis [40]. A meta-analysis of 64 studies showed that the definition of stroke—associated pneumonias varied widely [41].

The incidence of pneumonia post stroke has been reported to range between 1 and 44% [42, 43] and has been shown to increase mortality (threefold) and overall hospital care costs [42]. Two recent studies have looked at the utility of prophylactic antibiotics to reduce pneumonia. The STROKE-INF study [44] randomised patients with acute stroke and dysphagia to 7 days of prophylactic antibiotics (to be commenced within 48 h of stroke onset) or standard care and found no reduction in the incidence of pneumonia (OR 1.21, 95% CI 0.71–2.08, p = 0.489). The PASS study [45] investigated the effects of prophylactic ceftriaxone and found that this did not affect functional outcome at 3 months. While there was a significant reduction in infections overall, there was no effect on the incidence of pneumonia (OR 0.67 95% CI 0.39–1.15, p = 0.18). Therefore, current evidence does not support the use of antibiotic prophylaxis to prevent pneumonia. There are several validated risk scores which can help the clinician to identify patients at high risk of stroke-associated pneumonia [46–48]. Given the considerable morbidity and mortality, and the lack of benefit from prophylactic treatment, highlighting patients at high risk to allow early identification and treatment of established infection is important in the care of stroke patients.

Aspiration is a frequent cause of pneumonia post stroke, especially in patients with dysphagia. Dysphagia is seen in up to 50% of ischaemic strokes [49], although the reported incidence can vary between studies. Individuals suffering from dysphagia were three times more likely to develop pneumonia and this number increased to eleven times if they were shown to aspirate [50]. Often aspiration occurs silently (reported in up to 40%), that is, with few or no clinical signs. The presence of either dysphagia or a subsequent pneumonia is predictors of a worse clinical outcome [51]. An often neglected aspect of stroke treatment is oral care. Poor oral hygiene leads to proliferation of bacteria and debris in the oral cavity [52], which are liable to be aspirated causing respiratory tract infection [53]. This is particularly important in nasogastric tube fed patients in whom oral care can easily be missed.

Sleep apnoea is a common cause of intermittent nocturnal hypoxia after stroke, affecting up to 60% of patients [54]. This condition has also been shown to be a risk factor for future stroke and stroke mortality, if not appropriately treated. A few small studies have shown that nocturnal continuous positive airway pressure ventilation is feasible [55, 56], and can improve wellbeing in some stroke patients with sleep apnoea during the acute and rehabilitation phase, but compliance with the intervention is poor, especially in patients with delirium or cognitive impairment [57]. Obstructive sleep apnoea can cause or accentuate many traditional vascular risk factors, in particular hypertension [58] and atrial fibrillation [59], and has been shown to be an independent risk factor for stroke [54, 57, 60]. A review of the cohort of the Wisconsin Sleep Study found a significant association between sleep disordered breathing and stroke prevalence, the more severe the indices of sleep apnoea, the greater the risk [61].

Respiratory muscle function is also a potential cause of hypoxia either directly by associated muscle paralysis or as a result of a secondary infection. Several studies have shown (some in comparison to matched controls) a significant reduction in forced vital capacity, forced expiratory volume in one second, peak expiratory flow rate and maximal inspiratory and expiratory pressures [62–64], suggesting impairment in function of accessory respiratory muscles as well as the diaphragm. This may pave the way for treatment strategies aiming to improve respiratory muscle function [65].

Less common stroke complications resulting in hypoxia include pulmonary embolism, which despite its low incidence in most reported series (around 1%), is associated with increased in hospital mortality (31.5 vs 12.7% in a review of over 11,000 patients in the Registry of the Canadian Stroke Network), length of stay and severity of disability [66]. The risk of pulmonary embolism can persist for up to 4 weeks post stroke [67]. A review of a relatively small cohort of cryptogenic stroke patients found a significant incidence of silent pulmonary embolism (37%), but did not comment as to whether or not this led to a resultant hypoxic state [68]. Improvements in mechanical, pharmacological and therapy based regimes are the likely reason pulmonary embolism is now a relative rarity. Cardiac failure and very rarely neurogenic pulmonary oedema [69, 70] are among the other causes.

## Oxygen therapy for acute stroke

Oxygen treatment can be used to maintain normal oxygen saturation or to increase the oxygen saturation above normal in patients with acute stroke. The rationale for the latter is that blood with higher oxygen content may improve oxygen action in ischaemic brain areas [2]. When considering oxygen treatment it is important to weigh up potential adverse effects against benefits.

### Potential adverse effects of oxygen treatment after stroke

Oxygen treatment is not without side effects. Attachment to a wall delivery system as an inpatient restricts mobility in the acute phase and may represent an infection risk. In critical ill states or when bordering on the anaerobic threshold for exercise capacity, the body has several intrinsic systems to increase oxygen tension and deliver oxygen at the required rate in order to produce ATP and meet energy demands. One of the by-products of ATP formation is the formation of oxygen free-radical species, which, if not dealt with, can lead to cell apoptosis and developmental of tissue damage. In normal states the body has several intrinsic enzymes to neutralise free radicals by pairing them with so called donor electrons to form substances like oxygen or hydrogen peroxide which can then be efficiently removed. When high concentrations of oxygen are given this leads not only to increased oxygen delivery from red blood cells but also increased delivery via plasma. This then by-passes and overrides usual mechanism of clearance and is one the reasons tissue damage develops in inappropriately high concentrations of oxygen [71–73]. The cascade outlined above is only partially reversed during reperfusion, even though oxygen delivery has improved. Most of the clinical problems surrounding oxygen toxicity initially affect the lungs. High concentrations of oxygen may displace all nitrogen present in the alveoli and owing to the significant alveolar plasma gradient, the oxygen rapidly diffuses and dissolves into the plasma, effectively reducing the alveolar volume and leading to subsequent collapse. Hyperoxia may also impair mucilliary clearance and alter surfactant properties which may cause an ‘adhesive collapse’ [73, 74]. Neurological consequences outside of those described in the context of stroke include cerebral vasoconstriction, a by-product of excessive free radical formation, confusion, and seizures [73]. Oxygen toxicity more often occurs during use of high concentrations of oxygen or in hyperbaric conditions. In the clinical setting a stroke patient is exposed to, these are highly unlikely scenarios to occur.

### Recommendations from national and international stroke guidelines

A review of the most recent societal guidelines shows uniformity in the approach to oxygen therapy in acute ischaemic stroke. The Royal College of Physicians guidelines [75] advise use of supplemental oxygen only if oxygen saturation drops below 95% and is not contraindicated, and recommends no supplemental oxygen for saturations of 95% or above. The European Stroke Organisation [76] advises supplemental oxygen use for oxygen saturations of less than 95%. The American Heart Association/American Stroke Association Guidelines [77] advise that in the pre-hospital setting, oxygen supplementation to maintain oxygen saturations above 94% is reasonable and recommended for suspected stroke patients and that on presentation to hospital saturations should be continually monitored to watch out for hypoxia. This guidance is based on the American Heart Association post cardiac arrest guidelines [78] and thus the same advice applies to stroke patients. Again the guidelines do not support the use of hyperbaric oxygen therapy.

### Randomised controlled trials of supplemental oxygen in acute stroke

A plausible solution to aid the correction of cerebral hypoxia in stroke would be to provide supplemental oxygen therapy in the acute phase, potentially helping to correct or prevent many of the catastrophic cerebral changes that may occur. To date 6 randomised controlled trials have tested this hypothesis. A quasi-randomised study of routine oxygen supplementation within the first 24 h of acute stroke by Ronning and Guldvog [79] showed that routine oxygen treatment (3 L/min for 24 h) in unselected stroke patients did not reduce morbidity or mortality. Subgroup analyses suggested worse outcomes in patients with mild strokes treated with oxygen and a trend towards better outcomes in severe strokes (Scandinavian stroke scale score <40), but the study was not large enough to identify with certainty those who are likely to derive benefit. Oxygen saturation before or during treatment was not reported and it is therefore impossible to determine whether or not oxygen was ineffective because it failed to improve oxygen saturation or because of a genuine lack of effect on the ischaemic brain. A small study (n = 16) delivered oxygen at a rate of 45 L/min for 8 h, commencing with 12 h of stroke onset. Perfusion-diffusion mismatch on MRI showed that cerebral blood volume and blood flow within ischaemic regions improved in the hyperoxia. Neurological deficit improved at 4 h (during treatment), 24 h and at 1 week. By 24 h MRI of the brain showed reperfusion and (asymptomatic) petechial haemorrhages in 50% of hyperoxia treated patients and 17% of controls (p = 0.06). No long-term clinical benefit was seen at 3 months [80]. This study was too small to draw reliable conclusions, leading to a larger (unpublished) study by the same group (http://www.clinicaltrials.gov/ct2/show/NCT00414726?term=singhal&rank=1) which initially planned to enrol 240 patients, randomising to either room air or high flow oxygen (30–45 L/min for 8 h) within 9 h of acute stroke onset. After enrolment of 85 patients, the study was terminated early due to an imbalance of deaths favouring the control arm, though it is noted that the excess in mortality in the treatment group was not considered related to the treatment by an external blinded assessor. An Indian study [81] enrolled 40 patients within 12 h of an acute anterior circulation ischaemic stroke and a National Institute Stroke Scale of more than 4 to receive either 10 L/min for 12 h via face mask in the treatment group versus room air or 2 L/min to keep oxygen saturation above 95%. There was no significant difference in NIHSS, modified Rankin or Barthel index scores between the two groups. There was also no statistically significant difference between DWI lesion volumes in either group, though there was a trend towards smaller lesions in the treatment group.

In the Stroke Oxygen Pilot study [82], oxygen was given for 72 h and the dose was dependent on baseline oxygen saturation (2 L/min if the saturation was >93%, 3 L/min if the saturation was 93% or less). Initial results showed that the treatment regime increased oxygen saturation by about 2% in the treatment arm and this was associated with a small, but significant improvement in neurological recovery at one week. At 6 months [83] there was no statistically significant difference between the two groups, although there remained a small trend towards overall benefit with supplemental oxygen. This data led to the Stroke Oxygen Study (SO_2_S) [84], in which 8003 patients within 24 h of hospital admission with acute stroke were randomized 1:1:1 to receive either continuous supplemental oxygen, supplemental oxygen only at night (9pm–7am) oxygen, or no supplemental oxygen treatment for 72 h. This study has completed recruitment and is expected to report in 2016.

## Conclusion

Oxygen is a vital substrate to the continual function and survival of cerebral tissue. Rapid reduction in partial pressures can very rapidly lead to catastrophic and permanent cerebral injury and physical disability. Whilst evidence does not currently support the additional supplementation of oxygen to stroke patients, it remains important to prevent hypoxia in stroke patients by identifying and treating reversible causes rapidly. Results of the Stroke Oxygen Study will provide new evidence of whether prophylactic oxygen treatment can prevent neurological deterioration and improve recovery.
